# Relational Continuity of Chronic Patients with Primary and Secondary Care Doctors: A Study of Public Healthcare Networks of Six Latin American Countries

**DOI:** 10.3390/ijerph192013008

**Published:** 2022-10-11

**Authors:** Verónica Espinel-Flores, Gabriela Tiburcio-Lara, Ingrid Vargas, Pamela Eguiguren, Amparo-Susana Mogollón-Pérez, Marina Ferreira-de-Medeiros-Mendes, Julieta López-Vázquez, Fernando Bertolotto, Delia Amarilla, María-Luisa Vázquez

**Affiliations:** 1 Health Policy and Health Services Research Group, Health Policy Research Unit, Consortium for Health Care and Social Services of Catalonia, Avinguda Tibidabo 21, ES08022 Barcelona, Spain; 2 Escuela de Salud Pública Dr. Salvador Allende Gossens, Universidad de Chile, Independencia 939, Santiago de Chile, Chile; 3 Escuela de Medicina y Ciencias de la Salud, Universidad del Rosario, Cra 24 No. 63C-69, Quinta Mutis, Bogotá 11001, Colombia; 4 Grupo de Estudos de Gestão e Avaliação em Saúde, Instituto de Medicina Integral Professor Fernando Figueira, Rua Dos Coelhos No. 300, Boa Vista, Recife 50070-550, Brazil; 5 Instituto de Salud Pública, Universidad Veracruzana, Av. Dr. Luis Castelazo Ayala s/n Col. Industrial Ánimas, Xalapa 91190, Mexico; 6 Facultad de Enfermería, Universidad de la República, Avenida 18 de Julio 124, Montevideo 11200, Uruguay; 7 Maestría en Salud Pública, Centro de Estudios Interdisciplinarios, Universidad Nacional de Rosario, Maipú 1065, Rosario 2000, Argentina

**Keywords:** continuity of care, continuity of patient care, longitudinality, doctor–patient relations, primary health care, secondary health care, Latin America

## Abstract

Despite relational continuity (RC) with the doctor being key to care quality for chronic patients, particularly in fragmented healthcare systems, like many in Latin America (LA), little is known about RC and its attributes, particularly regarding specialists. Aim: We aim to analyse chronic patients’ perceptions of RC with primary (PC) and secondary (SC) care doctors, and record changes between 2015 and 2017 in the public healthcare networks of six LA countries. An analysis of two cross-sectional studies applying the CCAENA questionnaire to chronic patients (N = 4881) was conducted in Argentina, Brazil, Chile, Colombia, Mexico, and Uruguay. The dependent variables of RC with PC and SC doctors were: consistency, trust, effective communication, and synthetic indexes based on RC attributes. Descriptive and multivariate analyses were performed. Although the RC index was high in 2015, especially in PC in all countries, and at both levels in Argentina and Uruguay, low perceived consistency of PC and SC doctors in Colombia and Chile and of SC doctors in Mexico revealed important areas for improvement. In 2017 the RC index of SC doctors increased in Chile and Mexico, while SC doctors’ consistency in Colombia decreased. This study reveals important gaps in achieving RC with doctors, particularly in SC, which requires further structural and organisational reforms.

## 1. Introduction

Many Latin American healthcare systems are highly fragmented, leading—among other problems—to discontinuity in patient care, which ultimately affects the quality of care they receive. These problems are particularly pertinent in the case of patients with chronic conditions, who require care over long periods of time from various health services and professionals. They are therefore the most acutely affected by coordination problems across different levels of care, such as deficiencies in the transfer of clinical information between levels or disagreements over the clinical management of the patient [1,2].

In highly fragmented healthcare contexts, the analysis of relational continuity (RC) of patients with their health-service providers over time has become increasingly relevant to address the problems of cross-level care coordination [3,4]. Several Latin American governments have promoted health services network based on primary care which acts as coordinator of patient care throughout the care continuum of the health services [5]. RC is considered to be an essential component of primary care (PC), as it not only contributes to the effectiveness of care [5], but also helps to strengthen the role of PC as care coordinator. Various international and regional studies show an association between RC with the PC doctor/team and improved transfer and use of shared clinical information [5,6,7,8], clinical agreement with other care levels [5,7,8,9], accessibility to emergency services [10], and likelihoods of the patients returning to PC after referral to SC [5].

RC is also referred to as longitudinality [11] and has been the object of many theoretical frameworks [9,11,12]. This study applies the framework proposed by Reid et al. [12] that defines RC as the patient’s perception of an ongoing therapeutic relationship over time with one or more health professionals, and encompasses two dimensions: *(1) the consistency of the medical team*, which refers to the patient’s perception of always being attended to by the same professionals, and *(2) the provider–patient bond*, which refers to the patient’s perception of having a personal relationship with the health professional, based on feelings of belonging, trust, mutual understanding, patient loyalty and the professional’s sense of responsibility for their patient. RC can therefore only be analysed on one care level at a time since it refers specifically to doctor–patient interaction over the course of time. This differentiates it from continuity between care levels, of which there are two kinds—continuity of information and of clinical management.

To date, RC has mostly been studied in Europe and high-income countries mainly through quantitative studies based on patient surveys, or indirectly through the calculation of indicators based on clinical records. Most of the studies available focus on analysing just one attribute of RC in primary care [13,14,15,16,17], and very few on secondary care [18] or on both levels [19]. These generally study the association between consistency of the PC doctor (the number of visits to/by the same doctor) and care quality, the use of services, or the patient’s adherence to prescribed treatments [13,16,17,20]. Studies focusing comprehensively on the different attributes and dimensions of RC are highly scarce [10,12]. Consistency of the PC doctor is associated with a greater use of preventive services [20], shorter hospital stays [16,17], fewer visits to emergency services [15], and a lower risk of diagnosis of a chronic disease [13]. The few studies that have analysed consistency of care in SC report that patients seen by the same specialist place greater trust in their doctor, which in turn, is associated with better adherence to treatment [18].

Most of the Latin American studies available, some of which are comparative between countries [8,21,22,23], come mainly from Brazil [8,22,23,24], Mexico [21,22,25,26], and Colombia [8,21,22,23,27]. Likewise, very few of these study RC in a comprehensive way, with the majority focusing on analysing just one attribute of RC in PC—the consistency of the doctor [22], the doctor–patient bond [24,25], or communication with the doctor [27]—through either qualitative methods [27] or surveys of health services users [8,21,22,23,24,25] or people with social security in general [26]. Quantitative studies on chronic patients and SC doctors RC are extremely limited to date. There is only one study analysing it in patients with chronic diseases [25]. Some of the few studies that have comprehensively analysed RC through surveys show a negative perception of RC in PC—limited consistency and poor communication with the PC doctor is associated with perceived low quality of health systems in the region [22,26].

This study forms part of a wider research project, Equity-LA II [28], whose aim was to evaluate the effectiveness of interventions to improve cross-level care coordination and continuity in the public healthcare networks of six Latin American countries: Argentina, Brazil, Chile, Colombia, Mexico, and Uruguay [29,30]. Although the models vary, these countries have health systems that are segmented by population group according to socioeconomic or employment status [31,32], with a public subsystem and a private one. The public sector is financed by social security contributions and/or taxes. It encompasses at least one subsystem dependent on the ministry of health, which is decentralised to different levels of government (departments/provinces and/or municipalities) and is usually envisaged for the lower-income population and/or those without social security. In all five countries, the norms envisage health care organised by level of complexity, with PC as the entry point and the coordinator of patient care along the continuum of health services, and SC in a supporting role, requiring a referral from PC to access a specialist. This includes the introduction of different mechanisms with different levels of success in order to coordinate the management and support functions of the network, the access of patients across levels of care, to share patients’ clinical information (improved referral system), or to guarantee that health care is delivered in a coherent, complementary, and sequential way [33].

This study on RC and its diverse attributes in patients with chronic diseases complements the study of continuity of care across levels [30] with evidence on patient interaction with the primary care and the secondary care doctors over time, and changes in this throughout the years. Its aim is to analyse chronic patients’ perceptions of RC with PC and SC doctors, and record any changes between 2015 and 2017, in the public-healthcare service networks of six LA countries.

## 2. Materials and Methods

### 2.1. Design and Study Areas

An analysis was conducted of two cross-sectional studies in six Latin American countries, using the Questionnaire of Continuity of Care across Care Levels (CCAENA^®^) in face-to-face surveys of patients with a chronic disease who used the healthcare services of networks in 2015 and 2017.

Two healthcare networks were selected in each country, according to the following criteria: (a) provision of a continuum of health services, including at least PC and SC; (b) to a defined population; (c) in an urban environment of low/lower-middle socioeconomic status; and (d) willingness to participate in the study [7,30]. The health networks were: (1) Argentina, the south/south-western and north/north-western districts of Rosario; (2) Brazil, two micro-regions of Districts III and VII in Recife and the urban area of Caruarú; (3) Chile, two networks in the southern and northern metropolitan areas of of Santiago; (4) Colombia, networks of the southwest and south of Bogotá; (5) Mexico, networks of Xalapa and Veracruz; and (6) Uruguay, two networks in the eastern region [7,30].

### 2.2. Population and Study Sample

The study population consisted of healthcare services users aged over 18 who suffered from at least one chronic disease and had been seen in PC and SC in the previous 6 months for the same illness, whether acute or chronic. The sample size was estimated at 392 patients per health services network, 784 patients per country and per year, to detect a 10% variation in perceived RC in a bilateral contrast between networks and between years, with 80% power and a 95% confidence level [7,30].

Participants were selected through simple random sampling [7]. Due to the lack of administrative records of patients in some countries, the process was conducted in waiting rooms in primary care centres. The total sample comprised 4881 patients in 2015, and 4889 in 2017.

### 2.3. Questionnaire

The CCAENA^®^ Questionnaire was applied for data collection; its content was adapted and validated to the context of each country, and translated into Portuguese in Brazil. In each country, two pre-tests were carried out, and a pilot test conducted with 20 users, in order to check for interview pace, interviewer load, and acceptability and comprehensibility [7,28,34].

The final questionnaire had eleven sections: (1) health problem, (2–7) experience of continuity, accessibility, and perception of continuity between care levels, (8) factors related to continuity of care between care levels, and (9–11) insurance and sociodemographic data. This study utilised the data obtained in Section 8, which records patients’ perceptions of RC with SC and PC doctors.

### 2.4. Data Collection

Data were collected in face-to-face interviews conducted by specifically trained interviewers. The first survey ran from May to December 2015, except in Argentina (to April 2016), and Uruguay (to February 2016); the second ran from November 2017 to January 2018 in all countries.

To ensure the quality and consistency of the data, the interviewers were supervised in the field, all the questionnaires were checked, 20% of participants were re-interviewed at random, and the double-entry method was used to control inconsistencies during data entry.

### 2.5. Variables

The dependent variables of the study for RC in PC and SC were: (1) consistency of the doctor, (2) trust in the doctor, and (3) effective communication (the response categories, measured on a Likert scale, were dichotomised into “high level” for the responses always/often and “low level” for the responses hardly ever/never); and (4) RC index. Using the above three variables, a synthetic index was created to measure the degree of RC in PC and SC: each variable was given as score from 1 to 4, with a final maximum score of 12 points. Taking half of the maximum score as the cut-off point, the variable was categorised as “high” if the score was greater than 6.

The independent variables included were sociodemographic (sex, age, level of education, and length of residence in the area), morbidity-related (number of chronic diseases and self-rated health) and related to health services utilisation (use of health services of the network as regular source of care and use of out-of-network services). The stratification variables were country and year.

### 2.6. Analysis

A bivariate descriptive analysis of the sample was conducted to determine the distribution of the dependent and independent variables. To identify significant differences between years, the Chi-square test was used. To analyse changes in levels of RC between the two years, Poisson regression models with robust variance were estimated, obtaining prevalence ratios (PR) and their 95% confidence intervals (CI95%), adjusting for the independent variables (sociodemographic data, morbidity, and use of health services). Statistical analyses were conducted using Stata v.15 software [35].

### 2.7. Ethical Considerations

The development and execution of the project were conducted in adherence to all international and national conventions, legislation, and declarations, as well as the regulations on ethical issues, professional conduct, and data protection in all the countries concerned. Data-confidentiality and -privacy agreements were signed with all participating institutions, and informed consent was sought from all interviewees. The project was approved by an ethics committee in each country [28].

## 3. Results

### 3.1. Characteristics of the Sample

To facilitate the description of the sample, the names of the countries are used to refer to the study networks.

In 2015, the majority of the sample was female (ranging from 73.5% in Chile to 86.1% in Brazil), with a predominant age group of 40–64 years; however, this differed considerably between countries (from 48.5% in Uruguay to 72.2% in Argentina), except in Chile and Colombia, where the most common age group was 65 or over. Most participants had only completed primary education, with differences between countries (from 43.9% in Mexico to 61.1% in Uruguay), except for Chile, where the majority had completed secondary education, and Colombia, where the largest group consisted of users with no education/incomplete primary education. The vast majority had resided in the area 10 years or more (72.9% in Colombia; 90.1% in Uruguay); used the public network as their regular source of care (89.6% in Mexico; 96.4% in Chile); and did not use services outside the network (71.1% in Chile; 96.2% in Uruguay), except in Mexico. Approximately a third suffered from two chronic diseases (32% in Uruguay; 37.5% in Colombia), except in Argentina (49.8%), and in Brazil and Chile (the largest group was three or more diseases). Most perceived their own state of health as fair/poor/very poor (from 69.4% in Colombia to 84% in Brazil), except in Argentina and Uruguay, where these users were in the minority (Table 1).

In 2017, the percentage of women decreased in Chile and increased in Uruguay. The percentage of users aged 40–64 fell in Argentina and Mexico and rose in Uruguay. The percentage of participants with no education/incomplete primary education increased in Argentina, Brazil, and Chile and decreased in Mexico and Uruguay. A higher percentage of patients in Uruguay stated that they had resided in the area for 10 years or more. The percentage of participants who used the public network as their main source of health care rose in Brazil, Mexico, and Uruguay, as did the percentage of those who used services outside the network in Colombia, while the latter decreased in Mexico. In all countries except Chile and Uruguay, there was a rise in the percentage of participants who suffered from three or more chronic diseases, and in Argentina, Mexico, and Uruguay, there was also an increase in the percentage of people who described their self-rated health as fair/poor/very poor (Table 1).

### 3.2. Perception of Relational Continuity with the Primary Care and the Secondary Care Doctors

In 2015, a high degree of RC was perceived at both care levels in all six countries, but there were differences between levels and between countries, both in terms of indexes and attributes. The RC index was very high in both PC and SC, in all countries (highest scores: PC: 99.6% in Argentina and SC: 97.0% in Uruguay) with Chile scoring the lowest at both levels (PC: 84.2% and SC: 81.6%). Some differences were also observed between care levels in each country. The score was higher in PC than in SC in Brazil (PC: 96.3% and SC: 88.8%), Colombia (PC: 93.8% and 87.1%) and Mexico (PC: 95.8% and SC 87.6) (Table 2).

With regard to the attributes of RC, the level of *consistency of the doctor* was high in PC and SC in Argentina (PC: 97.6% and SC: 87.5%) and low at both levels of care in Chile (PC: 55.6%; SC: 55.2%). In the remaining countries, the consistency of doctors was greater in PC, except in Uruguay, where it was slightly higher for SC doctors (PC: 92.1% and SC: 93.8%). The lowest levels of consistency of SC doctors were found in Mexico (PC: 90.2% and SC: 55.4%) and Colombia (PC: 93.8% and SC: 46.3%).

Perceived *trust in the doctor* was also high in all six countries, at around 90% in both PC and SC, except in Chile where it was lower (PC: 75.8% and SC: 78.0%). Similarly, the perception of *effective communication* was high, particularly in Argentina and Uruguay, at both levels (around 90% and 95%, respectively) and lower in Chile (around 70%). The other countries showed similar results, with higher values in PC than in SC (Brazil: PC: 83.1% and SC: 77.2%; Colombia: PC: 80.8% and SC: 78.2%; and Mexico: PC: 88.2% and SC: 84.8%).

### 3.3. Changes in Relational Continuity with the Primary Care and the Seconday Care Doctors

The **RC index** in SC increased in 2017 in Chile (PR: 1.07; IC95%: 1.03–1.11) and in Mexico (PR: 1.06; IC95%: 1.03–1.10), where there was also an increase in the consistency of SC doctors (PR: 1.32, IC95%: 1.23–1.41) and in trust in SC doctors (PR: 1.05, IC95%: 1.01–1.08) (Table 3).

There was a drop, however, in the scores for *consistency of the SC doctor* in Colombia (PR: 0.75, IC95%: 0.66–0.84), and for *effective communication with the SC doctor* in Uruguay (PR: 0.96, IC95%: 0.94–0.98).

Although for the remaining attributes the changes were not statistically significant, it is worth noting that there was an increase in the level of perception of all the attributes of RC in PC and SC in Chile, Mexico, and Argentina (with the exception, in the latter, of effective communication).

## 4. Discussion

This study contributes to evidence on a little-explored topic, despite its relevance in the improvement of coordination and quality of care for chronic patients in Latin America (LA): relational continuity (RC) with the primary care (PC) doctor and the secondary care (SC) doctor in public healthcare networks of six countries. Unlike in other studies, chronic patients’ perceptions of RC with the doctor is analysed in a comprehensive way in both PC and SC, the latter of which has been less studied within and outside the region. To this aim, we used a common tool (the CCAENA Questionnaire) [34], which was validated and adapted to the six countries analysed, in order to measure the different attributes of RC. Moreover, the use of a common questionnaire allowed us to avoid the methodological limitations of international studies based on secondary data and to deepen the study in aspects considered relevant in international literature but not tackled in representative surveys; these include the regular source of health care, the use of out-of-network services, and particularly, the relational continuity of the patient with the secondary care doctor, which is less explored in the available literature.

The results show a high level of perceived RC with PC and SC doctors, both in the index of RC and its attributes, especially in Argentina and Uruguay. The perception of RC and the consistency of doctors at both levels was lower in Colombia and Chile, and in the latter, so was the perception of effective communication and trust in doctors in PC. In the comparison between years, only Chile and Mexico showed improvements in the RC index with SC doctors in 2017, and Mexico also improved in terms of consistency and trust in SC doctors.

### 4.1. High Level of Relational Continuity Perceived, Particularly in PC, with Important Areas for Improvement

The higher level of RC perceived in PC than in SC in most countries is in keeping with the organisational structure of public healthcare systems in LA, which establishes PC as the central axis of the system and as the care coordinator [33]. It has been shown that this model favours RC in PC as it promotes consistency, which leads to stronger doctor–patient bonds [8], especially for chronic patients [25]. However, the analysis of RC attributes also indicates room for improvement in the consistency of PC doctors, particularly in Chile and Colombia, which may point to deficiencies in the implementation of the model in the region [33,36]. The high level of RC perceived may also be related to the characteristics of the sample—mainly female, older age groups, and at least one chronic disease, which are individual factors that have been associated with perceiving a higher level of relational continuity [8,37,38].

The differences found between countries and care levels may be due to certain structural and organisational characteristics of the healthcare services that affect the continuity of care [30]. In Argentina, the high level of RC perceived with PC and SC doctors could be related to the fact that the PC-based care model has been established for a long time in the study networks [39] and that specialists in it have been allocated to PC centres for certain medical specialties [40]. The latter may have fostered consistency of SC doctors in the study networks of this country. In Uruguay, the high level of RC perceived at both care levels may be attributable to the smaller size of the study areas, which were located in smaller urban zones, and to the co-location of PC and SC doctors in the same health centres. These characteristics encourage doctors to get to know their patients [26,39], strengthening trust and mutual understanding, and creating a sense of responsibility in professionals towards their patients [3,41].

In contrast, the low levels in Chile and Colombia of consistency of PC and SC doctors, and—although to a lesser degree—of the other attributes, appear to be a result of the unstable working conditions of health professionals, who are contracted through a system that generates high staff turnover (over 60% of doctors in the study networks in Chile had temporary contracts as did almost 80% in Colombia, compared to 20% in the other countries [1]). The poor consistency of doctors makes it difficult to establish patient–provider bonds [8] and has significant repercussions on the care of chronic patients; it is related to a perception of low quality of care [21,22], poor access to emergency services [10], and a worse perception of continuity between care levels [7].

### 4.2. Changes in the Perception of RC in Chile, Mexico, and Colombia in Secondary Care

Changes were observed in the perception of RC in SC; although they require further analysis, this may be associated, firstly, with interventions implemented within the framework of the Equity-LA II Project to improve coordination between care levels in the networks analysed, and secondly, with contextual changes that took place during the study period.

Regarding the first point, following the implementation of joint meetings between PC and SC professionals via videoconference in Chile, an improvement was observed in patient perception of the transfer of clinical information and clinical management [29]; the dimensions of cross-level continuity of care, which, according to some studies, enhance aspects of RC such as trust in the clinical skills of doctors [42,43]; loyalty towards the doctor; and the continuity of clinical information [3].

On the second point, contextual changes in Mexico, such as the local government’s implementation of a referral and counter-referral system, had a similar effect on continuity between care levels in the study networks [30], which, alongside improvements in staff contracting in SC, may have had a favourable impact on trust and doctor consistency in SC. Conversely, in Colombia, the drop in the consistency of SC doctors, which already scored poorly in 2015, could be attributable to the implementation of the Integral Health Care Policy (PAIS) in the district, which promoted the restructuring of public health networks, leading to the merger of healthcare units and staff redundancies [44].

### 4.3. Limitations

Although the study was only conducted in two health services networks in each country, and one should be cautious in generalising the results to other regions of the study countries and of Latin America, it allowed us to identify critical elements in relational continuity. With regard to comparing two cross-sectional studies, we must bear in mind that these are descriptive studies and do not establish causality. The results are not considered to have been influenced by courtesy or interviewer bias, as data quality control strategies were used to rule out these potential problems. Due to space constraints, only a brief description of the healthcare systems of the countries participating in the study is provided in the introduction. However, the basic characteristics needed to understand the organisation of the health services were included.

## 5. Conclusions

This study provides comparative evidence between countries and between years on the RC of chronic patients with their PC and SC doctors in the public healthcare subsystems of six Latin American countries. The RC index and RC attributes were high in all six countries, particularly in PC. However, the differences observed between countries and between care levels—mostly in terms of consistency, trust, and effective communication with doctors—reveal that there are areas for improvement that require the kind of structural and organisational reforms which have been found to be key to improving quality of care for chronic patients. These include employment policies that create job stability for health professionals, which are essential in order to strengthen bonds with their patients, and thus, effectiveness of care; organisational changes to reinforce PC’s role as the point of entry and coordinator of the patient’s trajectory throughout the care continuum, and the supporting role of SC; and the implementation of mechanisms to improve the cross-level coordination of information and of clinical management, which are important not only for improving clinical coordination, but also for communicating better with patients and boosting their trust in the skills of the health professionals treating them. On a more general note, this study illustrates the complexity involved in the analysis of RC with PC and SC doctors, and the need to go into greater depth through studies that focus on various levels of care in the public healthcare networks of Latin America.

## Figures and Tables

**Table 1:** Sociodemographic, morbidity, and health services utilisation characteristics of the sample, by country and year. CCAENA 2015–2017.

	Argentina		Brazil		Chile		Colombia		Mexico		Uruguay	
	2015	2017	2015	2017	2015	2017	2015	2017	2015	2017	2015	2017
	n = 789	n = 784	n = 793	n = 827	n = 880	n = 893	n = 793	n = 802	n = 789	n = 790	n = 837	n = 793
**Variables**	*n (%)*		*n (%)*		*n (%)*		*n (%)*		*n (%)*		*n (%)*	
**Sex**												
Men	150 (19.0)	180 (23.0)	110 (13.9)	146 (17.7)	233 (26.5)	280 (31.4) *	182 (22.9)	153 (19.1)	198 (25.1)	166 (21.0)	215 (26.0)	154 (19.4) *
Women	639 (81.0)	604 (77.0)	683 (86.1)	680 (82.2)	647 (73.5)	613 (68.7) *	611 (77.1)	649 (80.9)	591 (74.9)	624 (79.0)	622 (74.3)	639 (80.6) *
**Age**												
18 to 39	161 (20.4)	150 (19.1) **	112 (14.1)	131 (15.8)	39 (4.4)	33 (3.7)	30 (3.8)	25 (3.1)	87 (11.0)	120 (15.2) *	40 (4.8)	85 (10.7) **
40 to 64	570 (72.2)	531 (67.7) **	481 (60.7)	518 (62.6)	397 (45.1)	360 (40.3)	380 (47.9)	391 (48.8)	452 (57.3)	426 (53.9) *	406 (48.5)	416 (52.5) **
≥65	58 (7.4)	103 (13.1) **	198 (25.0)	174 (21.0)	444 (50.5)	500 (56.0)	383 (48.3)	386 (48.1)	250 (31.7)	244 (30.9) *	387 (46.2)	292 (36.8) **
**Level of education**												
No education/incomplete primary	136 (17.2)	186 (23.7) *	299 (37.7)	325 (39.3) *	245 (27.8)	291 (32.6) *	368 (46.4)	354 (44.1)	336 (42.6)	285 (36.1) *	215 (25.7)	141 (17.8) **
Primary	458 (58.1)	422 (53.8) *	353 (44.5)	325 (39.3) *	262 (29.8)	297 (33.1) *	348 (43.9)	344 (42.9)	346 (43.9)	362 (45.8) *	511 (61.1)	522 (65.8) **
Secondary	190 (24.1)	169 (21.6) *	133 (16.8)	153 (18.5) *	332 (37.7)	270 (30.2) *	64 (8.1)	78 (9.7)	76 (9.6)	106 (13.4) *	108 (12.9)	124 (15.6) **
University degree/postgraduate	5 (0.6)	4 (0.5) *	5 (0.6)	17 (2.1) *	32 (3.6)	29 (3.3) *	12 (1.5)	24 (3.0)	31 (3.9)	36 (4.6) *	0 (0.0)	2 (0.3) **
**Length of residence in the area**												
≤10 years	159 (20.2)	151 (19.3)	139 (17.5)	151 (18.3)	105 (11.9)	95 (10.6)	215 (27.1)	186 (23.2)	86 (10.9)	93 (11.8)	52 (6.2)	51 (6.4) **
>10 years	627 (79.5)	630 (80.4)	650 (82.0)	671 (81.1)	775 (88.1)	796 (89.1)	578 (72.9)	616 (76.8)	703 (89.1)	696 (88.1)	754 (90.1)	737 (92.9) **
**Number of chronic diseases**												
One	393 (49.8)	342 (43.6) *	203 (25.6)	161 (19.5) *	145 (16.5)	149 (16.7)	236 (29.8)	160 (20.0) **	342 (43.4)	286 (36.2) **	386 (46.1)	363 (45.8)
Two	229 (29.0)	258 (32.9) *	220 (27.7)	241 (29.1) *	239 (27.1)	236 (26.4)	297 (37.5)	262 (32.7) **	288 (36.5)	270 (34.9) **	268 (32.0)	233 (29.4)
Three or more	167 (21.2)	184 (23.5) *	370 (46.7)	425 (51.4) *	496 (56.4)	508 (56.9)	260 (32.8)	380 (47.4) **	159 (20.2)	228 (28.9) **	183 (21.9)	197 (24.8)
**Self-rated health**												
Very good—good	433 (54.9)	377 (48.1) *	125 (15.8)	138 (16.7)	210 (23.9)	209 (23.4)	243 (30.6)	274 (34.2)	214 (27.1)	202 (25.6) *	465 (55.6)	380 (47.9) **
Fair—poor—very poor	353 (44.7)	404 (51.5) *	666 (84.0)	686 (83.0)	670 (76.1)	684 (76.6)	550 (69.4)	528 (65.8)	569 (72.1)	588 (74.4) *	354 (42.3)	412 (52.0) **
**Regular source of health care**												
Yes	747 (94.7)	759 (96.8)	716 (90.3)	800 (96.7) **	848 (96.4)	860 (96.3)	725 (91.4)	743 (92.6)	707 (89.6)	747 (94.6) **	761 (90.9)	772 (97.4) **
No	41 (5.2)	23 (2.9)	75 (9.5)	27 (3.3) **	31 (3.5)	32 (3.6)	68 (8.6)	59 (7.4)	78 (9.9)	42 (5.3) **	35 (4.2)	19 (2.4) **
**Use of out-of-network services**												
Yes	166 (21.0)	159 (20.3)	96 (12.1)	112 (13.5)	254 (28.9)	256 (28.7)	48 (6.1)	179 (22.3) **	426 (54.0)	364 (46.1) *	32 (3.8)	659 (7.4)
No	623 (79.0)	625 (79.7)	697 (87.9)	711 (86.0)	626 (71.1)	637 (71.3)	745 (94.0)	620 (77.3) **	363 (46.0)	426 (53.9) *	805 (96.2)	734 (92.6)

* *p*-value < 0.05. ** *p*-value ≤ 0.001 for differences between years. Some categories have missing values (<5%).

**Table 2:** Prevalence of perceived high level of relational continuity with primary care doctor and with secondary care doctor in the health services networks studied, by country and year.

	Argentina		Brazil		Chile		Colombia		Mexico		Uruguay	
	2015	2017	2015	2017	2015	2017	2015	2017	2015	2017	2015	2017
	n = 789	n = 784	n = 793	n = 827	n = 880	n = 893	n = 793	n = 802	n = 789	n = 790	n = 837	n = 793
**Variables**	*n (%)*		*n (%)*		*n (%)*		*n (%)*		*n (%)*		*n (%)*	
**Relational continuity with the primary care doctor**												
**Relational continuity index**	786 (99.6)	776 (99.0)	764 (96.3)	787 (95.2)	741 (84.2)	771 (86.3)	744 (93.8)	749 (93.4)	756 (95.8)	763 (96.6)	810 (96.8)	769 (97.0)
**Consistency of the doctor**												
When you make an appointment with the GP, are you always seen by the same doctor?	770 (97.6)	768 (98.0)	748 (94.3)	759 (91.8)	489 (55.6)	500 (56.0)	546 (68.9)	557 (69.5)	712 (90.2)	700 (88.6) *	771 (92.1)	719 (90.7)
**Trust in the doctor**												
Do you trust in the professional skills of your GP?	759 (96.2)	756 (96.4)	716 (90.3)	719 (86.9)	667 (75.8)	696 (77.9)	707 (89.2)	721 (89.9)	718 (91.0)	732 (92.7)	806 (96.3)	761 (96.0) **
**Effective communication**												
Does the GP give you enough information about your disease?	732 (92.8)	714 (91.1)	659 (83.1)	676 (81.7)	614 (69.8)	657 (73.6)	641 (80.8)	651 (81.2)	696 (88.2)	713 (90.3)	788 (94.2)	720 (90.8) **
**Relational continuity with the secondary care doctor**												
**Relational continuity index**	759 (96.2)	752 (95.9)	704 (88.8)	715 (86.5)	718 (81.6)	782 (87.6) **	691 (87.1)	682 (85.0)	691 (87.6)	736 (93.2) **	812 (97.0)	771 (97.2)
**Consistency of the doctor**												
When you make an appointment with the specialist, are you always seen by the same doctor?	690 (87.5)	698 (89.0)	577 (72.8)	564 (68.2)	486 (55.2)	512 (57.3)	367 (46.3)	281 (35.0) **	437 (55.4)	610 (77.2) **	785 (93.8)	740 (93.3)
**Trust in the doctor**												
Do you trust in the professional skills of the specialists that you visit?	738 (93.5)	742 (94.6)	669 (84.4)	691 (83.6)	686 (78.0)	733 (82.1) **	669 (84.4)	672 (83.8)	683 (86.6)	722 (91.4) *	805 (96.2)	755 (95.2)
**Effective communication**												
Do the specialists give you enough information about your disease?	711 (90.1)	690 (88.0)	612 (77.2)	625 (75.6)	619 (70.3)	678 (75.9) **	620 (78.2)	632 (78.8)	669 (84.8)	694 (87.9)	798 (95.3)	724 (91.3) **

The data displayed in this table show the distribution of the response categories “Always” or “Often”. * *p*-value < 0.05; ** *p*-value ≤ 0.001 in comparison between years.

**Table 3:** Changes in the perception of relational continuity with the primary care doctor and the secondary care doctor in the health services networks between 2015 and 2017, by country.

	Argentina	Brazil	Chile	Colombia	Mexico	Uruguay
	*2017/2015*	*2017/2015*	*2017/2015*	*2017/2015*	*2017/2015*	*2017/2015*
	*aPR (CI95%)*	*aPR (CI95%)*	*aPR (CI95%)*	*aPR (CI95%)*	*aPR (CI95%)*	*aPR (CI95%)*
**Relational continuity with the primary care doctor**						
**Relational continuity index**	0.99 (0.99–1.00)	0.98 (0.96–1.00)	1.02 (0.98–1.06)	0.99 (0.97–1.02)	1.01 (0.99–1.03)	1.01 (0.99–1.03)
**Consistency of the doctor**						
When you make an appointment with the GP, are you always seen by the same doctor?	1.00 (0.99–1.02)	0.97 (0.95–1.00)	1.01 (0.93–1.10)	0.99 (0.93–1.06)	0.97 (0.94–1.00)	0.98 (0.95–1.00)
**Trust in the doctor**						
Do you trust in the professional skills of your GP?	1.01 (0.99–1.03)	0.96 (0.93–1.00)	1.03 (0.97–1.08)	1.00 (0.97–1.03)	1.02 (0.99–1.05)	0.98 (0.97–1.00)
**Effective communication**						
Does the GP give you enough information about your disease?	0.99 (0.96–1.01)	0.97 (0.93–1.02)	1.05 (0.99–1.11)	1.00 (0.95–1.05)	1.02 (0.99–1.06)	**0.97** **(0.94–0.99)**
**Relational continuity with the secondary care doctor**						
**Relational continuity index**	1.00 (0.98–1.02)	0.97 (0.94–1.01)	**1.07** **(1.03–1.11)**	0.97 (0.94–1.01)	**1.06** **(1.03–1.10)**	1.01 (0.99–1.02)
**Consistency of the doctor**						
When you make an appointment with the specialist, are you always seen by the same doctor?	1.00 (0.97–1.04)	0.95 (0.89–1.01)	1.06 (0.98–1.14)	**0.75** **(0.66–0.84)**	**1.32** **(1.23–1.41)**	1.00 (0.98–1.02)
**Trust in the doctor**						
Do you trust in the professional skills of the specialists that you visit?	1.01 (0.99–1.03)	0.99 (0.95–1.03)	1.01 (0.97–1.05)	0.98 (0.94–1.02)	**1.05** **(1.01–1.08)**	1.00 (0.99–1.02)
**Effective communication**						
Do the specialists give you enough information about your disease?	0.97 (0.94–1.01)	0.98 (0.93–1.03)	1.03 (0.98–1.09)	0.99 (0.94–1.04)	1.03 (1.00–1.07)	**0.96** **(0.94–0.98)**

PR adjusted for sociodemographic variables (sex, age, length of residence in the area, level of education), morbidity-related variables (self-rated health), and variables related to health services usage (regular source of health care). Group of reference: year 2015.

## Data Availability

The data used in this study can be accessed at: http://www.equity-la.eu/es/publicaciones.php?t=IS (accessed on 5 July 2021).
